# Removable brace is as good as plaster cast after surgically treated distal radius fracture – a randomised controlled study of pain and wrist function

**DOI:** 10.1186/s13018-025-06097-0

**Published:** 2025-07-21

**Authors:** Johanna Blomstrand, Irén Sellbrant, Bengt Nellgård, Jon Karlsson, Monika Fagevik Olsén, Gunilla Kjellby Wendt

**Affiliations:** 1 Department of Orthopaedics, Institute of Clinical Sciences, Sahlgrenska Academy, University of Gothenburg, Gothenburg, Sweden; 2 Department of Anaesthesiology and Intensive Care, Institute of Clinical Sciences, Sahlgrenska Academy, University of Gothenburg, Gothenburg, Sweden; 3 Department of Health and Rehabilitation– Physiotherapy, Institute of Neuroscience and Physiology, Sahlgrenska Academy, University of Gothenburg, Gothenburg, Sweden; 4 Department of Occupational Therapy and Physiotherapy, Sahlgrenska University Hospital, Mölndal, Sweden; 5 Department of Anaesthesiology and Intensive Care, Sahlgrenska University Hospital, Gothenburg, Sweden; 6 Department of Orthopaedics, Sahlgrenska University Hospital, Gothenburg, Sweden

**Keywords:** Distal radius fracture, Hand therapy, Occupational therapy, Rehabilitation, Disability

## Abstract

**Background:**

A distal radius fracture (DRF) is one of the most common fractures and is often treated with open reduction and volar-plate fixation, followed by immobilisation with a cast. Both the type and length of immobilisation are still the subject of debate, and studies investigating a prefabricated brace as an alternative to a cast are lacking. The aim of this study was to evaluate and compare the outcomes in terms of patients’ perceived disability, pain and grip strength, in patients treated with conventional immobilisation using a plaster cast compared with a prefabricated, stable wrist brace. The hypothesis was that the brace would have equal effect compared to the traditional cast regarding perceived disability, pain and grip strength.

**Methods:**

The study is a randomised controlled study (RCT), in which 60 patients were allocated to two groups, to either a cast or a prefabricated brace, following surgery after a distal radius fracture. The patients were assessed at five follow-ups in terms of perceived disability, pain and grip strength.

**Results:**

The analysis of equivalence between the cast group and the brace group showed that the outcomes in the groups can be regarded as equivalent, since all the minimal clinical important differences (MCID) limits were outside the confidence range of differences (*p* < 0.05). This indicates that there is no difference in terms of a cast or brace in either patients’ perceived disability, pain or grip strength at the measured time points.

**Conclusions:**

A prefabricated, removable brace was shown to be an equally good choice for immobilisation as a cast, following surgery after distal radius fracture, in terms of patients’ perceived disability, pain and grip strength.

**Trial registration:**

FoU i VGR, project number: 228311, registered 13 June 2017, https://www.researchweb.org/is/vgr/project/228311. Retrospectively registered at http://www.clinicaltrials.gov (NCT03749174) on 21 November 2018 with information on inclusion start 3 September 2018.

## Background

A distal radius fracture (DRF) is one of the most common fractures in humans [1–5]. The injury is more common in women than in men, and it mainly affects people over the age of 50. The percentage of patients undergoing surgery after a DRF in Sweden is approximately 26% [6]. The most common surgical method is open reduction and volar-plate fixation followed by additional support using a cast for 2–3 weeks after surgery [7].

The surgical method of open reduction and volar-plate fixation allows early mobilisation, but the questions of both the type and length of immobilisation are still the subject of debate [8–10]. A traditional non-removable cast is still frequently used postoperatively, although some studies have argued that immobilisation after surgical treatment may not be necessary [9, 11]. It is of great importance that the plaster cast is properly applied and does not lead to unnecessary complications [12]. A non-optimal plaster cast can cause both stiffness and swelling of the fingers and lead to nerve entrapments, Complex Regional Pain Syndrome (CRPS) and pressure ulcers [13, 14]. To avoid complications, adjustments to or replacement of the plaster cast are often necessary when it is poorly fitted or due to postoperative swelling.

Previous research on postoperative immobilisation indicates no clinically important impact on patient-reported outcomes or radiological outcomes between a thermoplastic splint and a non-removable cast [15]. Moreover, it has previously been reported that no differences were observed in terms of functional results and complication rates in patients who did not receive postoperative immobilisation and also started wrist mobilisation at an early stage after surgery, although they consumed more analgesics compared with patients who were immobilised for two weeks [16]. Most previous studies comparing a cast and a splint focus on thermoplastic splints, but studies investigating a prefabricated brace as an alternative to a cast are lacking.

In a previous study from our research group [17], the quality of recovery in patients who was treated with a cast compared with a brace after distal radius fracture surgery were evaluated. This study measured quality of recovery in terms of pain, physical comfort, physical independence, psychological support and emotional state [18]. Patients who were treated with a prefabricated brace instead of a cast experienced more pain during the first 24 h after surgery, but the differences were neither statistically significant nor clinically relevant and a brace as postoperative immobilisation was therefore considered as a feasible option [17]. Whether the two interventions have any impact over time on patients’ perceived disability, pain and grip strength is still unknown.

The aim of this RCT was to evaluate and compare the outcomes in terms of patients’ perceived disability, pain and grip strength, between conventional immobilisation in a plaster cast for two weeks, compared with a prefabricated, stable wrist brace, for two weeks. The hypothesis was that the brace was equal to the traditional cast in terms of perceived disability, pain and grip strength.

## Methods

### Procedure

The study is a part of the “RADAR study”, a collaborative project between the Department of Anaesthesia, the Department of Orthopaedics and the Department of Occupational Therapy and Physiotherapy at Sahlgrenska University Hospital/Mölndal. The Regional Ethical Review Board in Gothenburg, Sweden, approved the study (ref 214 − 18) and it was registered at clinicaltrials.gov (NCT03749174) and at FoU in VGR (project number 228311). The study was conducted between 3 September 2018 and 15 June 2020 and the patients were consecutively included by a research nurse or anesthesiologist at Sahlgrenska University Hospital/Mölndal Hospital, Gothenburg, Sweden. The main study included 120 patients and further details about the project are described elsewhere [17, 19].

### Patients

Patients with a distal radius fracture scheduled for stabilising surgery were asked before surgery about participating in the study, based on the following inclusion criteria;

over 18 years of age, closed DRF classified as AO 23 A or C1 (Orthopaedic Trauma Association) [20], scheduled for surgical fixation with a volar plate, less than 17 days after the injury, maximum length of surgery less than 90 min. The exclusion criteria were multiple fractures, inflammatory diseases, dementia, severe psychiatric disorder or other cognitive dysfunction, ongoing drug and alcohol abuse, known local anaesthetic allergy, pregnancy and no fluency of the Swedish language. Fracture classification and operations was performed by experienced orthopaedic surgeons.

All the patients gave their written informed consent to participate before study inclusion. The patients were randomised to either a or b, see below:

- A dorsal plaster cast two weeks post-operatively followed by two weeks with a brace to use between mobilisation exercises (*n* = 30).
- A brace post-operatively, worn continuously for the first two weeks post-surgery, followed by two weeks of use between mobilisation exercises (*n* = 30).

**Fig. 1:**
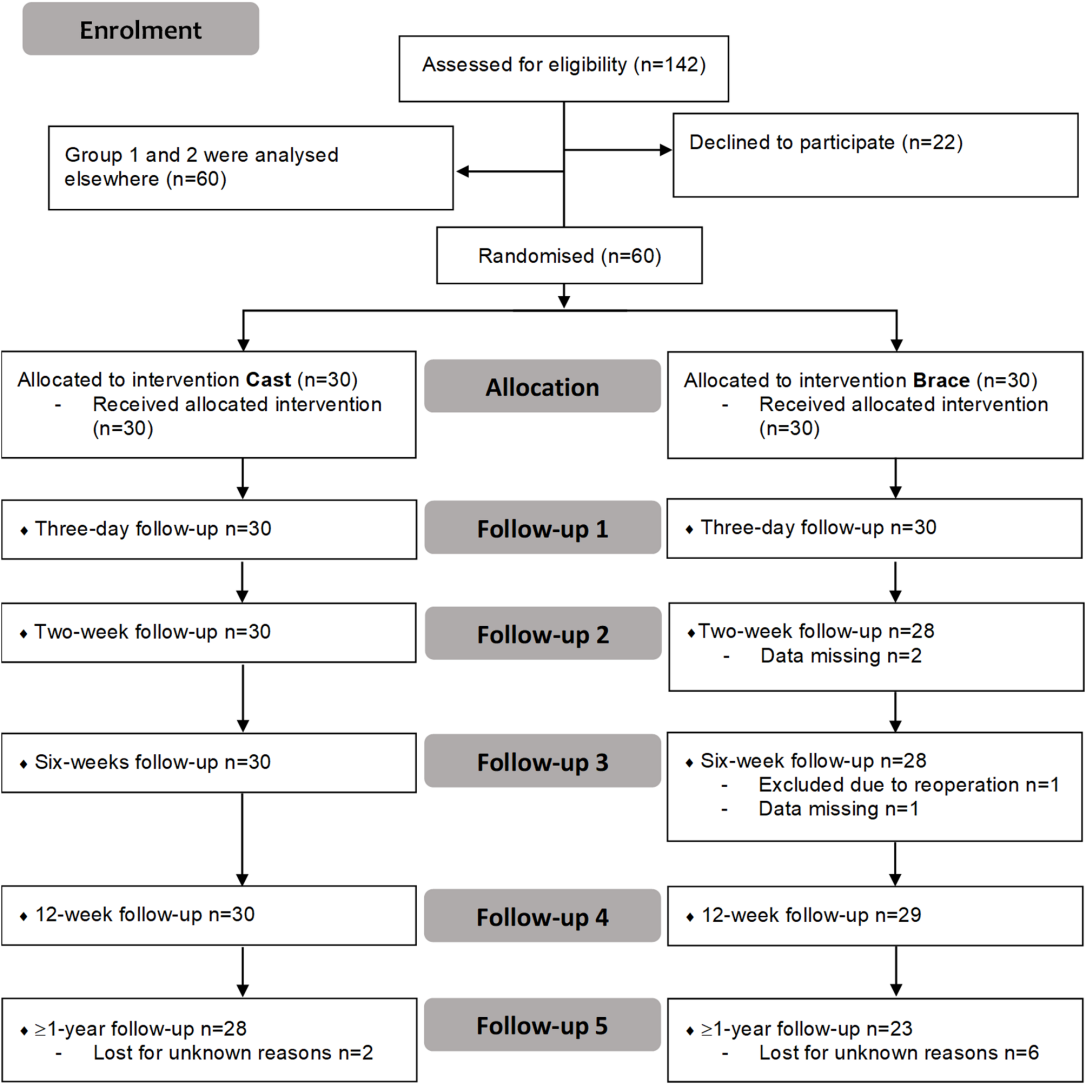
Flow chart. Groups 1 and 2 were included in the data collection in the overall project but were analysed in other parts of the project and presented elsewhere [17, 19]

The patients were randomised (1:1) using sequentially numbered envelopes with a random allocation sequence in two blocks, conducted by the same research nurse or anaesthesiologist before surgery [17]. Figure 1 shows the flow chart of the study.

The difference between the groups (28 versus 23) at the follow-up at > 1 year is not statistically significant.

The characteristics of the patients are presented in Table 1. The timepoints (median days after surgery) for the post-operative assessments were alike in both groups during the entire study period except for the last assessment, where the median days after surgery differed due to COVID-19 (cast group, a median of 480 days vs. brace group a median of 613 days after surgery, *p* < 0.05). There were no other significant differences between the groups.

**Table 1:** Characteristics of the patients (*n* = 60), mean (± SD) or number of patients (%)

	The cast group *n* = 30	The brace group *n* = 30	*p*-value
Age, years	60 (± 10) range 40–78	52 (± 16) range 18–74	0.09
Gender, female/male	26 (87%)/4 (13%)	23 (77%)/7 (23%)	0.32
Hand dominance Right/left	29 (97%)/1 (3%)	29 (97%)/1 (3%)	1.00
Injured hand Right/left	9 (30%)/21 (70%)	14 (47%)/16 (53%)	0.18
Injury to dominant hand Yes/no	10 (33%)/20 (67%)	13 (43%)/17 (57%)	0.43

All the patients received the same rehabilitation protocol according to current routines and consistent with local guidelines. After the open surgical repair using volar-plate fixation, all the patients were immobilised in a cast or a brace for two weeks, after which range of motion exercises were initiated. The brace used in the study was a stable, prefabricated brace with volar and dorsal aluminium rails (Wrist lacer, Camp Scandinavia AB).

### Assessments

Patients’ perceived disability, pain and grip strength were evaluated at five timepoints (three days and two, six and 12 weeks and ≥ 1 year after surgery), (Table 2).

**Table 2:** Overview of the assessments

	Three days	Two weeks	Six weeks	Twelve weeks	≥ One year
PRWE (disability)			X	X	X
Pain (NRS)	X	X	X	X	X
Grip strength (Jamar)			X	X	

Patient-reported outcomes in terms of perceived disability were assessed with Patient Rated Wrist Evaluation (PRWE) [21, 22], at six and 12 weeks and ≥ 1 year postoperatively and perceived pain, which was measured using a numeric rating scale (NRS) from 0 (no pain) to 10 (worst imaginable pain) [23], at three days and two, six and 12 weeks and ≥ 1 year postoperatively. The PRWE is a self-reported 15-item questionnaire designed to measure wrist pain and disability in activities of daily living and consists of questions related to pain and function, where function is divided into specific activities and general activities. The total score ranges from 0 to 100, where 0 is interpreted as a person with a well-functioning wrist with no pain, and 100 indicates major functional impairments and severe pain [21, 22].

Grip strength in both hands were measured by an occupational therapist, using a Jamar dynamometer [24] at six and 12 weeks postoperatively. The assessments were performed with the patients in a seated position, with the elbow flexed in 90 degrees and the wrist in a neutral position. The grip strength (kg) was measured three times, and the mean value was used for calculations. The primary outcome was PRWE at 12 weeks.

The assessments at three days, two, six and 12 weeks were performed as clinical visits, but due to the restrictions of COVID-19, the planned clinical follow-up of grip strength at ≥1 year had to be excluded and the questionnaires (PRWE and NRS) were sent home to the patients, who returned the questionnaires in a pre-paid envelope.

### Statistical methods

The sample size calculation was performed for the main study, where it was decided to include 30 patients in each group. Details are presented elsewhere [17]. No additional sample size calculation was made for this part of the study. However, to be able to detect differences in the PRWE of 11.5 points (minimal clinical difference (MCID) ± 15 points [25]) at 12 weeks, 27 patients per group were needed, with a power of 80% and a *p*-value of < 0.05.

The patients’ characteristics are presented using descriptive statistics as the mean, standard deviation and percentage. Differences in the proportion between groups in terms of categorical variables were evaluated using a chi-square test. Differences between the two groups regarding age and days after surgery were calculated using the Mann-Whitney U-test.

Changes over time between the different follow-ups in terms of disability, pain and grip strength are presented in median (Q1-Q3) and were calculated using Wilcoxon’s signed rank test. The results for the three variables at the five different timepoints are presented in median (Q1-Q3).

The above analysis was performed using SPSS version 28.0.1.0.

To assess the equivalence between the group differences for the primary outcome (PRWE), a Two One-Sided Test (TOST) of equivalence was performed to evaluate whether the predefined equivalence margin of ± 11.5 [25] was outside the confidence interval. Secondary outcomes were pain (assessed with NRS), with an MCID of 1.65 [26], and grip strength (kg), with an MCID of 6.5 [27]. As the data were not normally distributed, a Wilcoxon rank sum test for equivalence was performed. The analysis was performed using the R statistical software [28] and the TOSTER package (version 0.4.1) [29]. A *p*-value of < 0.05 was considered statistically significant.

## Results

Patient perceived function improved significantly between six and twelve weeks in both groups (*p* < 0.001), but from twelve weeks to one year, only in the cast group (*p* < 0.05). After a year, the median PRWE total score was 3 in both groups (Table 3), indicating a good overall outcome. Thirty-six patients, 60% (17 patients in cast group, 19 in brace group) estimated their pain (NRS) as 0 at six weeks, and 6 patients (4 in cast group, 2 in brace group) estimated their pain ≥ 3. From six weeks pain was scored 0 (median), indicating a low degree of pain. There was a significant decrease in pain between two and six weeks in the brace group (*p* < 0.01) and between six and twelve weeks in the cast group (*p* < 0.01). In terms of grip strength, the patients in both groups improved significantly between six and twelve weeks (*p* < 0.001) (Table 3).

**Table 3:** Results and changes over time for the three variables at the five different timepoints, median (Q1-Q3)

	Three days		Two weeks		Six weeks		Twelve weeks		≥1 year	
	Cast	Brace	Cast	Brace	Cast	Brace	Cast	Brace	Cast	Brace
Disability (PRWE total score)					30 (13–44) *n* = 30	20 (11–36) *n* = 28	11 (5–19)*** *n* = 29	11 (5–24)*** *n* = 27	3 (0–16)* *n* = 28	3 (0–21) *n* = 22
Pain (NRS)	2 (0–3) *n* = 30	1 (1–3) *n* = 30	1 (0–1) *n* = 30	1 (0–2) *n* = 28	0 (0–1) *n* = 30	0 (0–1)** *n* = 28	0 (0–0)** *n* = 29	0 (0–1) *n* = 28	0 (0–0) *n* = 25	0 (0–2) *n* = 20
Grip strength, kg (Jamar)					12 (8–16) *n* = 30	15 (11–18) *n* = 28	20 (18–26)*** *n* = 27	20 (16–28)*** *n* = 27		

***p < 0.001, **p < 0.01, *p < 0.05

P-values refers to significance within groups, over time

The study shows that both groups significantly improved with less perceived disability during rehabilitation; at six weeks, the median total score in the PRWE was 30 in the cast group and 20 in the brace group and, at 12 weeks, the median total score in the PRWE was 11 in both groups. According to descriptors of severity for PRWE scores by MacDermid et al. [30], this indicates mild and minimal disability respectively.

The analysis of equivalence between the results in the cast group and the brace group showed that the differences between the groups were so small that the groups can be regarded as equivalent, since all the MCID limits are outside the confidence range for differences, see Table 4 (*p* = 0.04-<0.001), except for PRWE at 6 weeks. This indicates that at most time points, there were no differences related to a cast or a brace in either patients’ perceived disability, perceived pain, or grip strength.

**Table 4:** Results of the analysis of equivalence between the cast group and the Brace group. Bold values indicate statistical and clinical equivalence

		Median of differences (cast group– brace group)	Lower CI	Upper CI	*P* value
**PRWE**	6 weeks Cast *n* = 30, Brace *n* = 28	5.0	-2.5	13.0	0.08
	12 weeks Cast *n* = 29, Brace *n* = 27	0.5	-3.5	5.0	**< 0.001**
	>One year Cast *n* = 28, Brace *n* = 22	0.0	-3.0	2.5	**0.002**
**NRS**	Three days Cast *n* = 30, Brace *n* = 30	0.0	-1.0	1.0	**< 0.001**
	Two weeks Cast *n* = 30, Brace *n* = 28	0.0	-1.0	0.0	**< 0.001**
	6 weeks Cast *n* = 30, Brace *n* = 28	0.0	-0.0	0.0	**< 0.001**
	12 weeks Cast *n* = 29, Brace *n* = 28	0.0	-0.0	0.0	**< 0.001**
	>One year Cast *n* = 25, Brace *n* = 20	0.0	-0.0	0.0	**< 0.001**
**JAMAR**	6 weeks Cast *n* = 30, Brace *n* = 28	-2.6	-5.0	0.3	**0.008**
	12 weeks Cast *n* = 27, Brace *n* = 27	0.3	-3.7	3.3	**0.005**

Note, that the *p*-values relates to the distribution and not to the exact values

During the first two weeks, there was a cast change in one patient, and minor cast adjustments in 26 of the 30 patients in the cast group.

## Discussion

The most important results in the current study indicated that a brace instead of a cast appears to be an equally good choice for immobilisation following DRF surgery in terms of patients’ perceived disability, pain and grip strength.

This study has focused on the effect regarding disability, pain and grip strength since it is often used in assessments and evaluation of distal radius fractures. However, there are other beneficial effects that have not been in focus of this study, but are crucial to discuss. It is possible to adjust the brace due to patients’ symptoms of increasing/decreasing oedema, which enables an optimal fitting of the brace. The fact that the brace is adjustable plays an essential role when there is a risk of oedema and could therefore contribute to preventing complications. From the health care perspective, the use of a brace could result in fewer visits to the clinic, since treatment with plaster casts requires a structured follow-up routine to ensure the fit of the cast. A cast with a bad fitting also needs to be changed or adjusted, often several times.

Previous results from another study from the same cohort have indicated that neither the quality of recovery nor unplanned healthcare contacts differ when using a brace instead of a cast following surgery with volar plating after DRF. Although no statistically significant time gain in terms of length of surgery was found in the brace group in the study, there was a small difference in the length of surgery in favour of the brace group and it is possible to speculate that using a brace would save time since the time for cast application is avoided [17]. It is also possible to speculate that a brace instead of a cast could be cheaper, as all patients still receive a brace at two weeks. Moreover, other previous studies have shown that, among other aspects, the type of postoperative immobilisation did not show any significant or clinically important impact on the final patient-reported outcome [15], which is in line with the results of the current study.

The planned follow-up at ≥ 1 year was converted to a follow-up by mail, due to COVID-19. The fact that the approach to data collection differed at the last follow-up may have affected the results, since the first four follow-ups took place on site at the hand unit while the last one took place at home. COVID-19 also affected the time frame for the study and caused many of the last follow-ups to be delayed, which explains the variation in terms of days after surgery. The variation in time so long after surgery is, however, not thought to be clinically relevant or to affect the patients’ perception of their outcomes at that time point, since most patients normally regain the majority of their range of motion, strength and function within three to six months [30].

The study is a randomised controlled study, which limits the risk of confounding factors, although the immobilisation methods were not blinded to the occupational therapists who treated the patients. Several occupational therapists were also involved in the data collection, which could be interpreted as a limitation, although everyone was carefully informed before the project started and followed the same protocol throughout the project. This fact can also be interpreted as a strength, as it reflects real-world clinical work. The patients were mostly treated by the same occupational therapist during the rehabilitation period.

Study limitations include the restriction of the sample to only AO type A and C1 distal radius fractures, limiting the generalizability of the findings to other fracture types. Furthermore, we did not assess patient satisfaction or discomfort related to the immobilisation method, nor did we consider rotational limitations associated with the use of a brace or cast. Additional limitations were the lack of data on the number of outpatient visits, including any extra visits required for cast adjustment, as well as the absence of evaluation of immobilisation-related complications. We also did not assess patient compliance with the assigned immobilisation method, which could have provided valuable insights. Lastly, due to the constraints imposed by the COVID-19 pandemic, radiographic measurements comparing intraoperative and final healed wrist positions were not obtained. This would have been of particular value for evaluating long-term outcomes.

This is a sub-study of a larger study, and the sample size calculation was made for the main study. With the power analysis presented in the methods section in mind, we included approximately this calculated number of patients in the study. Regardless, there are no trends in the data that implies that the results would be different if the groups were to be larger.

## Conclusions

A prefabricated, removable brace is an equally good choice for immobilisation as a cast, following DRF surgery, in terms of patients’ perceived disability, pain and grip strength.

## Data Availability

The datasets generated during and/or analysed during the current study are available from the corresponding author on reasonable request.
